# A Query Language for Exploratory Analysis of Video-Based Tracking Data in Padel Matches

**DOI:** 10.3390/s23010441

**Published:** 2022-12-31

**Authors:** Mohammadreza Javadiha, Carlos Andujar, Enrique Lacasa

**Affiliations:** 1 ViRVIG Research Group, Computer Science Department, Universitat Politècnica de Catalunya-BarcelonaTech, 08034 Barcelona, Spain; 2 Complex Systems in Sport Research Group, Institut Nacional D’Educacio Fisica de Catalunya (INEFC), University of Lleida (UdL), 25192 Lleida, Spain

**Keywords:** sports science, racket sports, video-based analysis, player tracking, sport analytics, data analysis, data visualization

## Abstract

Recent advances in sensor technologies, in particular video-based human detection, object tracking and pose estimation, have opened new possibilities for the automatic or semi-automatic per-frame annotation of sport videos. In the case of racket sports such as tennis and padel, state-of-the-art deep learning methods allow the robust detection and tracking of the players from a single video, which can be combined with ball tracking and shot recognition techniques to obtain a precise description of the play state at every frame. These data, which might include the court-space position of the players, their speeds, accelerations, shots and ball trajectories, can be exported in tabular format for further analysis. Unfortunately, the limitations of traditional table-based methods for analyzing such sport data are twofold. On the one hand, these methods cannot represent complex spatio-temporal queries in a compact, readable way, usable by sport analysts. On the other hand, traditional data visualization tools often fail to convey all the information available in the video (such as the precise body motion before, during and after the execution of a shot) and resulting plots only show a small portion of the available data. In this paper we address these two limitations by focusing on the analysis of video-based tracking data of padel matches. In particular, we propose a domain-specific query language to facilitate coaches and sport analysts to write queries in a very compact form. Additionally, we enrich the data visualization plots by linking each data item to a specific segment of the video so that analysts have full access to all the details related to the query. We demonstrate the flexibility of our system by collecting and converting into readable queries multiple tips and hypotheses on padel strategies extracted from the literature.

## 1. Introduction

### 1.1. Padel Essential Characteristics

**Figure 1:**
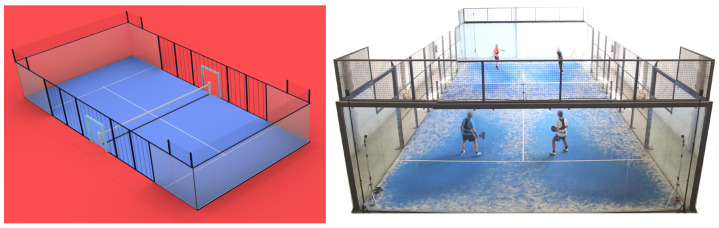
A padel court is substantially smaller than a tennis court. The court is enclosed by plexiglass walls and a metal mesh.

Padel is a modern racket sport that is becoming increasingly popular worldwide [1,2,3]. Although padel shares some features with squash and tennis, it also has important distinctive characteristics [3,4]. As in tennis, the court (20 × 10 m) is divided by a central net, but one of the most important differences between padel and tennis is that the padel court is delimited by walls (glass and metal mesh) except for two openings at the outer side of the net (Figure 1). Players are allowed to return the ball after it bounces on the walls, which decreases the technical ability to begin practicing it, and results in a varied range of shot types [5]. Furthermore, the serve in padel requires the player to bounce the ball and hit it below the hip. Unlike tennis, padel is essentially a doubles game, and thus partners need to collaborate to return the ball and to disturb the opponents to win the point. At the same time, padel is an open sport that requires constant decision-making, and the technical-tactical behavior of the player has an enormous and endless margin for evolution. This explains why the analysis of padel matches is so attractive and justifies the need of tools allowing researchers and coaches to access the data to understand and study them.

### 1.2. Video-Based Tracking in Sport Science

In the last few years, we have witnessed a significant development of deep learning techniques, which currently offer unprecedented results in video-based object detection, recognition, and tracking. Although the interest in player tracking [6] and video analysis for sports [7] is not new, convolutional neural networks have greatly improved the accuracy of computer vision tasks. These advances have opened new opportunities for video-based performance, tactical and biomechanical analyses in sports.

In this paper we focus on video-based spatio-temporal data in padel. The use of computer vision techniques in racket sports (such as tennis) has been studied extensively for tasks such as player tracking [8,9], ball tracking [10,11,12,13], shot recognition [14], content-based retrieval [15], virtual replays [10], and automatic annotation [16]. Concerning the positional analysis of padel players from a single video, besides direct observation [17], different approaches perform video analysis from zenithal [18,19] or nearly zenithal cameras [20]. For the de-facto camera standard in padel matches, state-of-the-art techniques provide per-frame court-space player positions [21], despite distinctive features of padel such as enclosing glass walls, inter-player occlusions, and occlusions by the mesh panels and structural elements.

Player positions can be obtained in multiple ways. *Detection* algorithms can recognize and locate instances of multiple persons in an image, which are highlighted through a collection of enclosing rectangles [22,23,24,25,26]. *Image segmentation* algorithms (e.g., [25,27,28]) label each pixel of the image with the ID of the predicted class (e.g., person/background). Finally, *pose estimation* methods estimate the location of keypoints (e.g., head, shoulders, hip, feet) of detected people. Current pose estimation methods can be classified into top-down and bottom-up methods. *Top-down* methods (e.g., [23,29,30,31]) first detect person instances, and then their individual joints, whereas *bottom-up* methods (e.g., [32,33]) detect first all keypoints in the image, and then group keypoints into person instances.

Although many pose estimation methods can operate on still images, when processing videos the best accuracy is achieved by combining them with tracking methods. State-of-the-art tracking methods such as DeepSORT [34], PoseFlow [35] and Tracktor++ [36] have been shown to perform very well on sports data. For a recent comparison of 3D pose estimation and tracking in sports videos, we refer the reader to [37].

### 1.3. Structuring Tracking Data

The deep learning advances discussed above greatly automate the task of generating per-frame annotations of the matches, which can include essential data about the players (positions, poses), the ball (position, bounces), and the play itself (shots, winning points, scores). All these data can be extracted automatically or semi-automatically from a single video of the match, and then put in tabular form for its posterior analysis. Table 1, Table 2 and Table 3 show examples of how raw video-based data on points, shots, and frames can be structured into tables. The video-based tracking nature of the data is reflected by the fact that sometimes teams and players are identified by their position on the video (e.g., top left or TL player) rather than by name.

Unfortunately, the advances in getting player/ball tracking data from sports videos are not on par with the development of interactive data analysis and exploration tools enabling non-IT professionals to perform complex queries on such datasets. The focus of this paper though is not on getting the data but rather on providing a high-level language to facilitate their analysis.

We can apply traditional data analysis approaches to analyze the data, but these approaches do not allow non-experts to retrieve or analyze complex spatiotemporal relationships in a compact, readable way. In the context of video-based padel data, we have identified two major limitations in current data analysis approaches, which are discussed below.

### 1.4. Retrieving Data about Specific In-Game Situations from Tabular Data

Sports analysts, coaches, and professional players often make strategy recommendations (player positioning, synchronized actions, best technical actions for a given scenario) that might or might not be sufficiently supported by empirical evidence, or that might apply only to certain circumstances (e.g., they may apply to professional players but not to amateurs). Some samples of typical recommendations for padel are:

**E1**  “Players should try to win the net zone as much as they can; it is easier to score a point from the net zone than from the backcourt zone”.
**E2** 
“An effective way to win the net zone is to play a lob”.
**E3** 
“When a player is about to serve, his/her partner should be waiting in the net zone, at about 2 m from the net”.

The availability of tracking data from padel matches opens great opportunities to provide empirical support to such recommendations, to refute them, to quantify their impact, or to analyze under which circumstances they apply (men’s matches vs. women’s matches, professional vs. amateur, adult vs. child players, right-handed vs. left-handed). Similarly, coaches and sports analysts might be interested in comparing the decision-making processes of a player with those of elite players.

Following the example sentences above, there are many options to exploit the data. For **E1**, we could estimate the conditional probabilities *P(winning the point*|*net zone)* and *P(winning the point*|*backcourt zone)* by computing the relative frequencies of winning points for the two conditions, from a sufficiently large and representative set of matches. If matches are conveniently labeled, we could also compute and compare these probabilities for different match categories (e.g., indoor vs. outdoor).

Regarding **E2**, we could follow a similar approach and estimate *P(winning the net*|*lob)*, that is, the probability that a team wins the net after playing a lob. If large datasets on elite players are available, we could also measure the relative frequency of lob shots compared to other types of shots, and assume that elite players take the best technical actions most of the time.

Concerning **E3**, we could plot the court-space position of server partners, and analyze e.g., whether the distance $d_{n}$ to the network and the distance $d_{w}$ to the lateral wall are normally distributed. If so, we could compute a simple Gaussian model for these variables, e.g., $d_{n}∼N\mu_{n},\sigma_{n}^{2})$, where the parameters $\mu_{n}$, $\sigma_{n}^{2}$ can be estimated from the data.

These types of analyses are certainly possible using tracking data in tabular form. However, to the best of our knowledge, no specific languages/tools have been reported to transform the raw tabular data from a collection of matches into the data that are relevant to the problem at hand. In other words, we are not aware of any high-level domain-specific language facilitating the filtering and retrieval of such padel data. The same lack of tools also applies to tennis and other racket sports. As a consequence, such analyses must be based on conventional tools, for example through manual counting, spreadsheets (filters, transformations, formulas), or computer programs operating on the tabular data [38].

Referring to the previous examples **E1**–**E3**, let us consider what queries could retrieve data to support, refute or qualify them. The following queries (in plain English form) could be useful for this task:

**Q1** 
Retrieve all points, distinguishing by the winning team and the zone of the hitter player.
**Q2**  Retrieve all lob shots with an additional column indicating whether the players could win the net zone or not.
**Q3** 
Retrieve all frames immediately after a serve, along with the court-space position of the server’s partner.

Although all these queries can be implemented, for example, in a spreadsheet, depending on the query complexity these tasks might require a considerable effort. Let us suppose that, starting from a table similar to Table 3, we wish to retrieve the position of the server’s partner in the 2 s immediately after each serve. We start with a spreadsheet example, as this is a tool commonly used by sports analysts. First, we should identify, for each frame, which of the four players is the server’s partner. Since this information is missing on the Frames table, we could add a column “Server partner” that, given a frame number, retrieves the game it belongs to, and the server’s partner for that game. A vertical lookup function (vlookup in most spreadsheets) could help with this task. Then, we should remove all frames outside the 2-s window after a serve. Again, this would require adding more columns (with non-trivial lookup functions) to compute the time offset between each frame and the serve. Additional functions will be required to select the server’s partner position out of the four player. Finally, we could sort the data by time offset, remove the rows with an offset above the 2-s threshold, select the (new) column with the network distance, and plot/analyze the results.

The spreadsheet example above already shows the different drawbacks of this approach. First, it requires non-trivial transformations of the data: adding new columns, using lookup functions (just computing column offsets for the result is error prone), sorting the data (or setting up filters/dynamic tables). Second, this approach lacks scalability. When new data come in, many of the steps above have to be repeated for each match. Third, it lacks flexibility: if our definition of “net zone” changes (e.g., it is moved 50 cm away), this would require extensive changes in the spreadsheets. Finally, it lacks readability, as the computation and filter formulas are spread over the cells.

It can be argued that, as a preprocess, we could enrich the tabular data to simplify these kinds of analyses. As we shall see (Section 5.3), padel concepts are so diverse that this approach would only benefit the simplest queries. Notice that the query example above (based on **E3** and **Q3**) is relatively simple. Queries involving sequences of events (e.g., drive-lob-volley) further hinder the required transformations.

High-level programming languages provide convenient data structures and methods to analyze tabular data. Python has a relatively smooth learning curve compared to other programming languages, and it is extensively used for data analysis. Pandas is a well-known Python package that provides a DataFrame class, which is essentially a convenient representation of tabular data. Similar data structures and methods are available in other languages (such as R, Octave and MatLab). These languages provide a convenient way to transform and query tabular data, but the resulting code is often too complex and unreadable to be usable by coaches and sports professionals. Some queries do admit a very simple expression. For example, retrieving all serves in Python using pandas can be as simple as: serves = shots[shots[‘Shot type’]==‘Serve’], where shots is the input DataFrame (Table 2), and serves is the output DataFrame. Unfortunately, other types of queries are harder to write (Section 8.4). Many queries require combining data from multiple tables, which at the end require using either lookup functions or, in the case of DataFrames, different types of joins [39] (inner joins, outer joins, left joins, right joins). Join operators are a concept from database theory and relational algebra that requires data-retrieval skills. However, even mastering join operators, queries involving sequences of events (e.g., “lobs followed by a defensive smash and then a volley in the net zone”) require additional operators that are usually too complex for people with no background in relational algebra.

### 1.5. Extracting Essential Information without Missing Relevant Details

A video of a padel match contains valuable information that can be hardly captured in tabular formats, such as the exact poses throughout the execution of a technical action, verbal communication between players, and non-verbal communication (e.g., gestures when rivals try to avoid playing to a particular player). Although sometimes we wish to analyze the data through abstraction, an essential part of the exploratory analysis is to interactively examine the details of specific situations. In traditional motion data analysis, the tabular data are often explored independently from its video source, with some notable exceptions such as [38]. This prevents sports analysts from performing a deep analysis of the data. For example, after finding that some player often loses the point after a particular technical action (e.g., an off-the-wall smash), a coach might want to retrieve all segments of the video where this situation occurs. Our solution to this problem is to provide interactive plots where, whenever possible, data items include links to the part of the video where they occur. If appropriate tools are used, this kind of plot greatly speeds-up the analysis of context information (body orientation, feet positioning, impact location and timing) that can provide valuable insights to improve the player’s performance.

### 1.6. Contributions

The main contribution of this paper is the definition (and a free and open-source prototype implementation) of a domain-specific query language to define queries on video-based data from padel matches. Domain-specific languages (DSLs) are tailored to a specific application domain and thus provide important advantages over general-purpose languages (GPLs) in such domain [40]. In particular, we propose a domain-specific language embedded in a GPL (more precisely, a Python API). Our language has greater expressive power, and ease of use, thus enabling writing queries in a simple, compact, flexible, and readable way.

Furthermore, and although not the main focus of the paper, we propose a collection of interactive visualization tools to visually explore the output of such queries. A major novelty is that data items are seamlessly connected to video segments so that a precise analysis of specific technical actions is integrated into the exploratory analysis process.

For evaluating the power and expressiveness of the query language, we have collected multiple statements about padel strategies (tips, comments, pieces of advice, hypotheses…) from different published sources (books, papers). We discuss how to design queries to support, refute or analyze these hypotheses, and show how these queries can be written using our query language. The Supplementary Material shows a demonstration of our query system running on a Jupyter notebook.

## 2. Design Principles for the Query Language

Our ultimate goal is to develop a query language allowing sports analysts to perform exploratory analysis on video-based tracking data in the most efficient way. More precisely, we wanted the language to outperform traditional data analysis approaches in the following aspects:

**Expressiveness** We wish the language to support complex queries, combining arbitrary conditions on player positions, poses, distances, shot attributes, court zones, timing, scores, sequences, and any other fact in the tabular data or that can be derived from it (such as speed, acceleration, motion paths).
**Compactness** Queries (even complex ones) should require little space (e.g., a few lines of code).
**Expandability** Analysts should be able to easily extend the language to incorporate their vision of fuzzy concepts. For example, different analysts might want to define court zones using different criteria and reuse these concepts in queries. When it comes to processing data, many concepts in padel (e.g., “forced error”, “good lob”) need to be defined precisely, and the concrete definition might vary among analysts, or depend on the player profiles (professional vs. amateur). Once these concepts are defined, they should integrate seamlessly into query definitions.
**Easy to write** We wish sports analysts to be able to write new queries, or at least be able to modify existing examples to suit their needs.
**Easy to read** We wish sports analysts to be able to understand the queries after a brief introduction to the main concepts of the language.

Among all the criteria above, we prioritized expressiveness. As a consequence, we decided that the domain-specific query language had to adopt the form of an internal DSL, embedded in Python. We thus defined an API (Application Programming Interface) for the Python language. Compared to external DSLs, which require an automated code generator to transform it into programming language code, embedded DSLs fully benefit from an already existing programming language. This approach considerably lowers the entry barrier for users already knowing the GPL. It also allows IDEs (e.g., Jupyter Notebooks, frequently used for data analysis) to recognize the syntax of the DSL and thus provide full support to code completion, syntax highlighting, and error checking. Another advantage is that it simplifies the description of the grammar and its implementation, since the DSL reuses the grammar and the parser of the GPL.

## 3. Design of the Query Language

### 3.1. Domain Analysis

The design of a DSL starts with an analysis of the application domain to create a feature model [41]. A Feature Diagram (FD) describes graphically the main features of the domain along with its dependencies. The FD is usually represented as a tree where nodes represent domain features and arcs connecting the nodes determine the relationships between them. Close dots denote mandatory nodes, whereas open dots represent optional nodes. The following subsections discuss the FDs of our application domain.

#### 3.1.1. Domain Analysis of a Video-Recorded Padel Match

**Figure 2:**
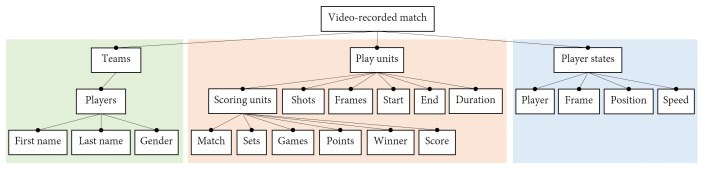
Feature diagram of a video-recorded padel match.

**Figure 3:**
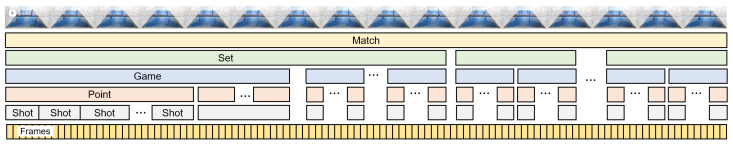
Hierarchy of temporal play units in a padel match.

The FD of a video-recorded padel match is shown in Figure 2. The diagram shows the main features of the basic questions on padel match: *who* (teams, players), *when* (temporal play units), and *how/where* (position, speed, type of shot). As shown in the diagram, a padel match involves two *teams* composed of *players* that can be identified by their names. On a temporal plane, we can distinguish four *scoring units* in padel (as in tennis): *match*, *set*, *game* and *point*. Each of these scoring units have a *winner* (winning team) and results in a *score* update. Other smaller temporal units are the *shots* and the *frames* of the video. Although a shot is an event, we can also consider the temporal unit between one shot and the next; similarly, we will use the term *point* to refer to the temporal unit (often called rally) between a serve and the moment where one of the teams wins the point. Altogether, a video-recorded padel match can be broken down into these play units, as illustrated in Figure 3. As such, each play unit has a *start* time, an *end* time, and a *duration*.

On a spatial plane, computer vision and other sensing techniques allow the automatic tracking of the players’ positions during a match. Therefore, the video-recorded match also contains the collection of *player states*, one for each *frame*, describing the *position* of the player within the court. These positions allow the computation of new parameters, such as speed, acceleration, distances to different court elements, and distance to the partner or opponent players.

**Figure 4:**
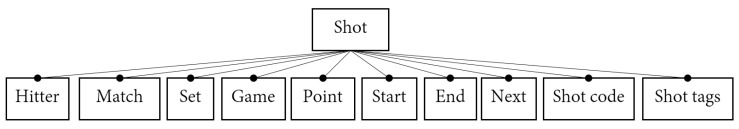
Feature diagram of a shot.

We can further analyze each of these concepts. For the sake of brevity, we only focus on shots, since they represent arguably the most relevant technical actions in padel. Figure 4 shows the FD of a shot in padel. A shot starts with some player (*hitter*) hitting the ball. The shot belongs to a specific match, set, game, and point, as shown in Figure 3. Shots have a *start* frame (when the player hits the ball) and an *end* frame (corresponding to the next shot, or end of the rally). Finally, shots have a collection of properties (such as the shot code) that, due to their complexity, will be discussed later in Section 7.

#### 3.1.2. Domain Analysis of Queries about Padel Matches

**Figure 5:**
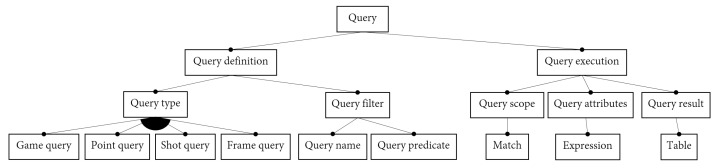
Feature diagram of a query on a padel match.

The FDs above describe the main features of a padel match from the point of view of videos annotated semi-automatically. Our language though deals with *queries* about these matches. Therefore queries are also an essential part of our application domain. Figure 5 shows the FD of such a query. We can distinguish two main parts: the *query definition* and the *query execution*.

An important remark is that we only consider queries whose output is represented as tabular data, because most data analysis software use this representation. The research literature on padel often considers parameters from the point of view of *sets*, *games*, *points*, and *shots*. We should also add *frames* since videos can be annotated automatically on a per-frame basis. Therefore we consider queries returning a table whose rows belong to one of these concepts. Thus we support the *query types* shown in Figure 5. Besides the query type, a query definition includes a *query filter*, that is, a predicate that represents some condition on the chosen concept. For example, a *Shot query* includes a predicate that determines whether a shot satisfies the intended condition.

Once a query has been defined (for example, to retrieve specific shots from a given player), the query can be executed on multiple matches. The *query execution* has three sub-features. The *query scope* is the match or collection of matches to be searched; the *query attributes* are the columns we wish to have in the output table, and the *query result* is the actual output table. The output table will contain one row for each item (e.g., shots) of the query scope that satisfies the query filter, and one column for each query attribute.

### 3.2. Query Language Syntax

The FDs reveal important concepts of the application domain and their structure. The next step is to define the DSL syntax and semantics. This can be achieved formally or informally [40]. In the latter case, the specification is given in natural language through a set of illustrative DSL examples. Since our DSL is embedded in the Python language, instead of describing its abstract syntax, we directly show the translation of our application domain concepts into components of the Python language (see Table 4) and show illustrative examples in the next sections.

The first block of Table 4 refers to concepts related to video-based padel matches (Figure 2). All these concepts are translated into Python classes or class properties. The second and third blocks refer to queries about matches (Figure 5). Essentially, a query definition corresponds to the definition of a Python function, and a query execution translates into a function call. We use Python decorators to simplify queries as much as possible. A decorator is a simple mechanism of the Python language for defining higher-order functions, that is, functions that take another function and extend its behavior. This mechanism is convenient because it moves a large part of the boilerplate code from the query definition to the internal implementation of the API.

## 4. Components of a Query

**Figure 6:**
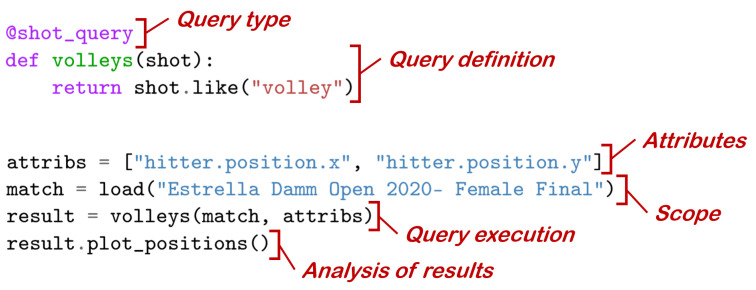
Simple query using the proposed API, along with its main components.

A query in our language requires four major components (see Figure 6), which are described below.

**Query type** The output of all our queries is a table with the retrieved data (more precisely, a QueryResult object that holds a Pandas’ DataFrame). The *Query type* refers to the different types of queries according to the expected output (that is, the type of the rows in the output DataFrame). Table 5 shows the query types supported by our language. From now on, we will use the generic word “item” to collectively refer to the entities (points, shots, frames…) that will form the rows of the output.
**Query definition** The query definition is a Boolean predicate that establishes which items should be retrieved (e.g., all shots that match a specific shot type). In our language, this takes the form of a decorated Python function that takes as input an object of the intended class (e.g., a Shot object if defining a shot query) and returns a true/false value. These predicates act as filters that discard items for which the predicate evaluates to false, and collect items for which the predicates evaluate to true. The output table will contain as many rows as items satisfy the predicate.
**Attributes** This refers to the collection of attributes we wish for every item in the output table (that is, the output table will have one column for each attribute). For example, for every smash, we might be interested only in the name of the player, or also in its court-space position, or just the distance to the net. One of the key ingredients of our language is that attributes are arbitrary Python expressions, with the only condition that they should be able to evaluate correctly from the item. For example, a shot has attributes such as hitter (the player that executed the shot), frame (the frame where the shot occurs), etc. Attributes can be simple expressions such as shot.hitter or more complex ones such as shot.next.hitter.distance_to_net < 3.
**Scope** Once we have specified the elements above, we might want to execute the query on different collections of matches. The scope is the collection of matches that will be searched for items fulfilling the predicate.

The separation of the different query components allows analysts to maximize reusability. For example, the query definition in Figure 6 filters all the shots to select only volleys. Later on, we can reuse this definition with different attribute collections, depending on what data about each volley we wish to analyze. Some attributes that make sense for this query definition include hitter.last_name, hitter.position.x, hitter.position.y, and frame.frame_number, just to give a few examples.

## 5. Key Features of the API

### 5.1. Navigation through Method Chaining

**Figure 7:**
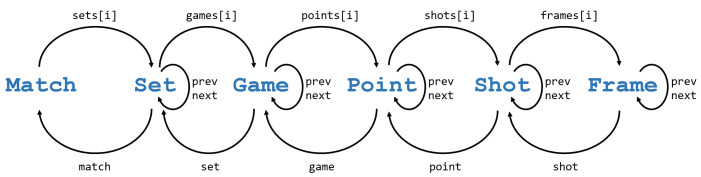
Main temporal units (Python classes) in our API, and methods/properties connecting them.

We exploit the hierarchical and sequence relationships of the main concepts introduced in Section 3.1 to provide a natural way to navigate through related concepts. Figure 7 shows the methods that can be used to get access to related elements. Consider, for example, the classes Point and Shot. The following Python expressions illustrate the use of these methods; for each expression, we indicate the item it provides access to:

point.shots[i] *# the i-th shot of a point*
shot.point     *# the point the shot belongs to*
shot.next     *# the next shot within the point (or None if last shot)*
shot.prev     *# the previous shot within the point (or None if serve)*

The relationships among the other classes in Figure 7 work the same way. These operations can be chained arbitrarily to get access to the data we are interested in. This is especially useful in query definitions (since the Boolean function gets as a parameter a single object, for example, a Shot) and it is also useful for attributes:

shot.hitter                              *# player that executed the shot*
shot.prev.hitter                         *# hitter of the previous shot*
shot.next.next.hitter.distance_to_net    *# for two shots ahead, distance to net of the hitter*
shot.point.winner                        *# team that won the point the shot belongs to*
shot.point.game.winner                   *# team that won the game the shot belongs to*

Although not shown in Figure 7 for simplicity, methods that allow traversing the hierarchy upwards can skip intermediate classes. For example, the expression

frame.shot.point.game.set.match.gender

can be written simply as frame.match.gender. Although implementation details are discussed in the Appendix A, we wish to note that the methods above are implemented as Python properties (using the @property decorator). Therefore instead of writing

shot.next().next().frame()

we can omit the parentheses and write

shot.next.next.frame

which is a bit more compact. Since query definitions require read-only access to all these objects, we consider that using properties instead of methods is safe.

### 5.2. Binding Players to Frames

When writing query definitions, we have observed that many times one needs to get attributes of a player (e.g., his/her position) at a specific moment during the game. In our API, the Player class represents personal information about the player (such as first name, last name and gender). As a consequence, if for example shot.next.next.hitter returns a Player object, his/her position will not be directly accessible. For the sake of compactness, we allow Player objects to refer to a particular frame of the video. In that case, we say the player object is *bound* to a specific frame. Methods returning a Player object return a temporary object that is bound to a specific frame (whenever this makes sense). For example, in a volley-drive-smash sequence, shot.next.next.hitter.position correctly gets the position of the player that plays the smash.

### 5.3. Tag Collections

Literature about padel tactics refers to many varied concepts attached to the central classes in Figure 7. For example, the following is just a small sample of concepts related to a shot: “unforced error”, "from attack zone", "defensive shot”, “half volley”, “drop shot”, “bring (the ball) back”, “backspin”, “overhead”, “at waist level”, “block”, “cross court stroke”, “long shot”, “soft shot”, and “double wall”. It is clear that including all these concepts as properties of a Shot class is not feasible nor convenient, since many of them are somehow fuzzy and can be defined in multiple ways (e.g., how much time, from the ball bounce to the shot impact, separates a half volley from other types of shot). Therefore, we decided to provide a minimalist set of properties for major classes, but let the classes be expandable so that analysts can add, at runtime, the concepts they wish. We achieve this expandability through two mechanisms, described below.

First, all these classes have a tags property that represents a dynamic set of tags, where a tag is just a string that encodes some predefined (e.g., “serve”) or user-defined (e.g., “flat serve”, “double fault”) attribute. These classes provide a like method to check whether an object has some tag. For example, shot.like(“volley”) is a shorter form of “volley”in shot.tags, and shot.like(“cross-court volley”) is equivalent to “cross-court”in shot.tags and “volley” in shot.tags. We have observed that this syntax is very compact and readable when searching for specific frames, shots, or points: shot.like(“winning smash from-defense-zone by-galvan”).

Second, we provide a simple method to define new tags, that is, a function to filter the objects that should include a user-defined tag. The syntax is nearly identical to that for query definitions (a Boolean function that gets as a parameter an item); the only difference is the function decorator, @shot_tag vs. @shot_query:

*# Define a new tag describing a volley near the net*
@shot_tag
def attack_volley(shot):
        return (shot.like(“vd”) or shot.like(“vr”)) and shot.hitter.distance_to_net < 1.5
*# Add the tag to a match*
attack_volley(match)
*# Query definitions can now use the new concept*
@shot_query
def attack_volley_after_return_of_serve(shot):
  return shot.like(“attack_volley”) and shot.prev.like(“return”)

In the example above, “vd” and “vr” refer to drive volley and backhand volley, resp. [5]. The new concept can be added to a match by simply invoking the function on a match or list of matches so that queries can use the new concept. The Python function decorator deals with the necessary code to traverse the match items (in the above case, shots) to check whether the new tag has to be inserted in the tag set.

## 6. A Complete Example

Before describing the API in more detail, here we briefly discuss a complete example, including also a first analysis of the query results. Lines beginning with # are just comments.

*# Define the query*

@shot_query

def attack_drive_volley(shot):
    return shot.like(“vd”) and (shot.hitter.distance_to_net < 5)

*# Define the attributes*

attribs = [“tags”, “frame.frame_number”, “hitter.position.x”,
      “hitter.distance_to_net”, “hitter.last_name”]

*# Execute query on a match*

match = load(“Estrella Damm Open’20 - Women’s Final”)
result = attack_drive_volley(match, attribs)

*# Analyze the results*

result.analyze()
result.plot_positions()

**Figure 8:**
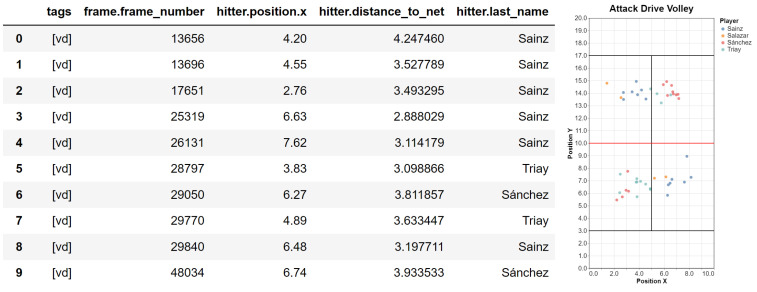
Output from the execution of a query on drive volleys less than 5 m away from the net. We only show the first rows of the output DataFrame.

The example is analyzing the position of the players when playing a drive volley less than 5 m away from the net. The query execution returns a QueryResult object, which provides some essential visualization methods. Figure 8 shows the output of the analyze and plot_positions methods.

## 7. Classes and Properties

Since our domain-specific language reuses the syntax of Python language, we need to describe the Python classes provided by the API. These classes provide properties and methods that can be used both in query definitions and query attributes. We first discuss classes representing temporal concepts (from Match to Frame), and then the Player class.

### 7.1. Match, Set, Game, Point, Shot, Frame

**Figure 9:**
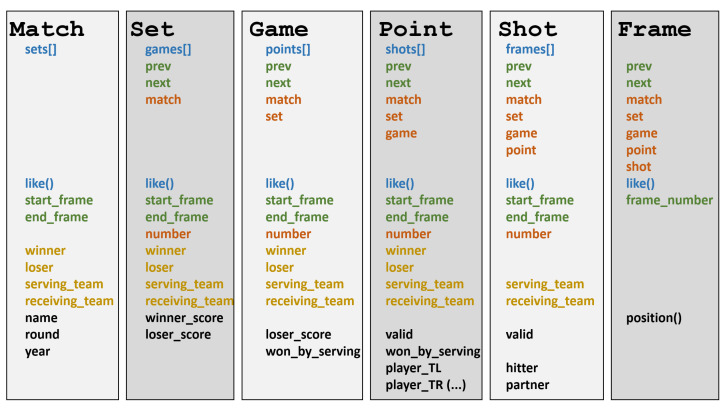
Main classes and methods in our API. Colors indicate coherent methods across classes.

Figure 9 summarizes the main classes of the API, along with their most relevant methods and properties. For the sake of conciseness, we only discuss the main classes and a subset of their attributes. See the accompanying implementation for full details. Additionally, for some properties with long names, we show an abridged version of it.

Referring to Figure 9, about one-half of the methods refer to the hierarchical and sequence relationships already discussed in Section 5.1. As already mentioned, these methods allow analysts to navigate through the different elements, as in shot.point.prev.winner, that given a shot, gets the team that won the preceding point.

Besides these hierarchical and sequence relationships, all these classes have a tags attribute (not shown in Figure 9) that contains a set of strings encoding specific concepts about the class (Section 5.3). The presence of a tag can be checked with the like method, as in the expression shot.like(“serve”). All these classes have associated a time interval of the video, represented either as start_frame and end_frame properties, or just a frame_number. Sets, Games, Points, Shots also have a number with their position within their parent class. For example, for the first set of a match, set.number==1. Winning and losing teams are available for all temporal units for which this makes sense (Match, Set, Game, and Point). Points include a valid attribute to distinguish e.g., net shots.

### 7.2. Shot Types

Tags can also include non-string objects, provided that they *behave* as strings. This feature of Python is called Duck Typing, which is a concept related to Dynamic Typing. We fully benefit from this feature and provide a ShotType object that allows shot types to be represented by multiple equivalent strings (codes or full names, and in multiple languages). Our current prototype uses the shot classification proposed by [5]. Table 6 lists these shots, along with multiple strings that can be used to refer to them. For example, a query could use either shot.like(“vd”) or the longer form shot.like(“forehand volley”).

### 7.3. Player

**Figure 10:**
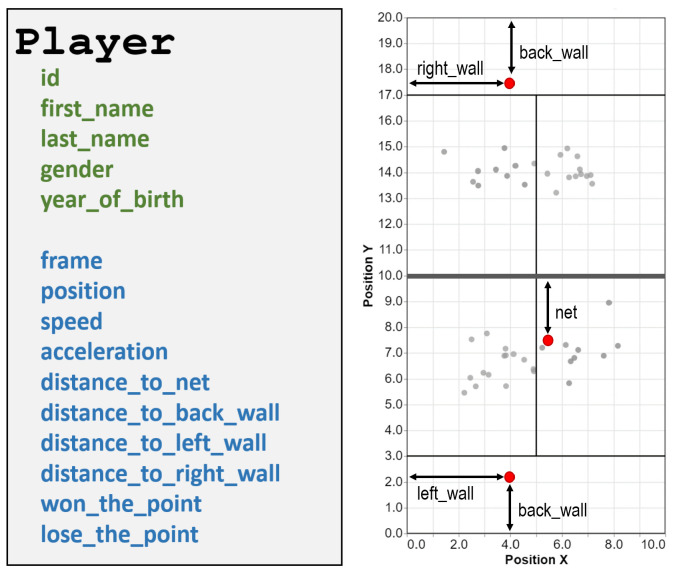
**Left:** Main methods of the Player class. The methods in green are available for any Player object, whereas those in blue are available only for Player instances bound to a particular Frame. **Right:** Reference system for players’ positions and distances.

Figure 10 summarizes the main methods of the Player class. Since most methods are self-explanatory, here we only explain position, speed, and acceleration methods. These three methods return a 2D point (position) or a 2D vector (speed, acceleration) with (x, y) coordinates/components. Figure 10 shows the global coordinate system for the global position of the players within the court. We also provide relative distances to major court elements (net and walls). Notice that, for these relative distances, the reference element is taken with respect to the player. For example, in distance_to_right_wall, the right wall is defined with respect to the player; the left wall for the players of one team is the right wall for the opponents and vice versa.

## 8. Evaluation

We evaluated the expressiveness of our query language by selecting many different statements about padel from the literature, and translating them into (informal) plain English queries and then into query definitions.

### 8.1. Test Dataset

As a test dataset, we used tabular data obtained by annotating semi-automatically a public padel match: the women’s final round of World Padel Tour’s Estrella Damm Open 5 July 2020, Madrid Arena. The video is publicly available https://youtu.be/7s55wB9dR78 (accessed on 1 December 2022). The position of the players within the court was obtained automatically following [21], in particular combining cascade detectors [27] based on ResNeXt [42] with a HRNet [32] keypoint estimator. These methods achieved accuracy on par with those from human annotators, with more than 98% of the estimated positions within a 30 cm error tolerance with respect to ground truth, for players on the bottom half of the court. Positions are less accurate for players on the top half of the court (due to error amplification by the camera perspective) and players with both feet in the air. Frame numbers are approximate since we used the *YouTube Player API Reference for Iframe Embeds* https://developers.google.com/youtube/iframe_api_reference (accessed on 1 December 2022) to play the video from specific time stamps. Since this section aims to evaluate the effectiveness of the query language, rather than drawing conclusions on the player’s performance, *we removed about one-half of the points from the dataset before running the queries.*

### 8.2. Test Statements

We list below the statements about padel strategies (mostly general observations and recommendations) we collected from padel coaches, books, papers, and websites. In Section 8.3 we will design and write queries that could be used to support, refute or analyze these.

S1 “One generally volleys cross-court” (source: [43])
S2 “A very effective volley is a fast, down-the-line volley to the opponent’s feet” (source: [43])
S3 “An interesting aspect of women’s padel is that the game speed is close to a second and a half, to be exact 1.37 s” (source: [3], referring to a 2020 sample of padel matches).
S4 “The serve should be a deep shot, targeted towards the glass, the T, or the receiving player’s feet” (source: basic padel tactics).
S5 “When a player is about to serve, his/her partner should be waiting in the net zone, at about 2 m from the net”. (source: basic padel tactics).
S6 “The serve is an attempt to seize the initiative for the attack, therefore the serving team tries to maintain the initiative by approaching the net”. (source: [5]).
S7 “Players should try to win the net zone as much as they can; it is easier to score a point from the net zone than from the defense zone” (source: basic padel tactics).
S8 “The (physiological) intensity developed during the practice of padel is close to that experienced in the practice of singles tennis (…). The real demands are different. This is probably due to the shorter distance covered by padel players in their actions. An aspect that can be compensated by a greater number of actions compared to tennis, due to the continuity allowed by the walls”. (source: [5]).
S9 “An effective way to win the net zone is to play a lob” (source: [43]).
S10 “The data corroborate one of the maxims that surround this sport: first return and first volley inside, implying that no easy point should be given to the opponents” (source: [5]).

### 8.3. Queries

Each of the statements above can be translated into multiple queries. We show below some plausible options, both in natural language and using our DSL.

#### 8.3.1. S1: “One Generally Volleys Cross-Court”

This statement can be addressed with the following query:

**Q1** 
“Retrieve all shots that are volleys (either forehand or backhand)”.

According to the shot classification we adopted (Table 6, we wish to include both forehand volleys (vd) and backhand volleys (vr). Since we foresee that many queries might use this “volley” concept, we are going to define it as a shot tag:

@shot_tag
def volley(shot):
    return shot.one_of(“vd,vr”)
 
volley(match) *# add “volley” tag to shots in this match*

Now, we will retrieve all volleys with a simple query. Notice that we can now use like(“volley”) within the query definition:

@shot_query
def volleys(shot):
    return shot.like(“volley”)

We will estimate the volley direction by computing the vector from the player’s position to the receiver player’s position, so we need to include as attributes the position of shot.hitter and shot.next.hitter players. We will also add additional attributes for plotting the data (such as player’s last name, and shot direction encoded as an angle):

volley(match)
attribs = [“frame.frame_number”, “hitter.position.x”, “hitter.position.y”, “hitter.last_name”,
      “next.hitter.position.x”, “next.hitter.position.y”, “angle”, “abs_angle”]
q = volleys(match, attribs)
 
q.plot_directions(color=‘angle’)
q.plot_distribution(density=‘angle’, extent=[-30,30], groupby=‘player’)

**Figure 11:**
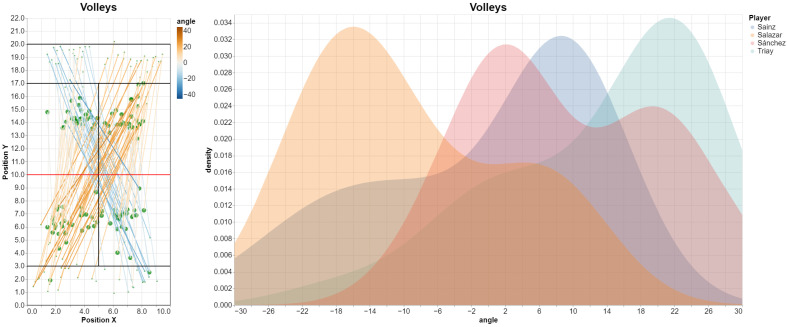
Direction of the volleys for the women’s final, with shots colored by angle (**left**). We show as well the estimated density distribution of the *volley angle* variable, for the four players (**right**).

Figure 11 shows the resulting plots. For each segment, the larger dots represent the volley origin, and the smaller dots the volley destination (estimated from the position of the opponent player that returned the ball).

Notice that the query above can be extended easily to look for specific types of volleys: for example, volleys played at a certain maximum distance from the net, after a specific type of shot, or from a specific player:

*# Volleys shot from less than 4 m from the net*
@shot_query
def volley_from_attack_zone(shot):
    return shot.like(“volley”) and shot.hitter.distance_to_net < 4
 
*# Volleys after a drive shot from the opponent*
@shot_query
def volley_after_drive(shot):
    return shot.like(“volley”) and shot.prev.like(“drive”)

#### 8.3.2. S2: “Effectiveness of a Fast, Down-the-Line Volley to the Opponent’S Feet”

The translation of this statement into a query (**Q2**) is straightforward:

**Q2**  “Extract all forehand or backhand volleys whose duration is below some threshold (1 s) and that are down-the-line”.

There are several ways for measuring a down-the-line volley; we will use the angle of the shot path, but we could use alternative criteria, such as the x position of the hitter and returning players. We will also use the “volley” tag introduced above. From now on we will omit the definition of the attribute list attribs if it can be easily inferred from the output plots:

@shot_query
def fast_down_the_line_volley(shot):
    return shot.like(“volley”) and shot.duration < 1 and shot.abs_angle < 8
 
q=fast_down_the_line_volley(match, attribs)
q.plot_directions(color=‘angle’)
q.plot_directions(color=‘winning’)

**Figure 12:**
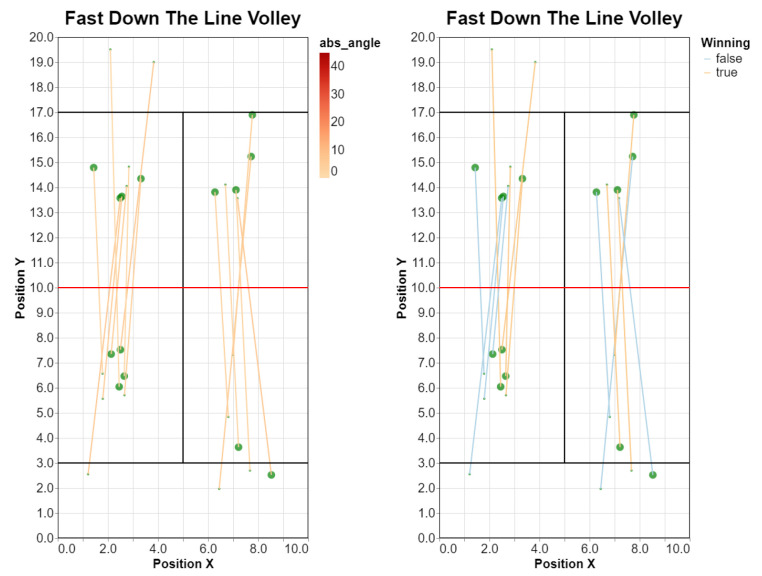
Direction of fast, down-the-line volleys for the test match, with shots colored by angle (**left**) or depending on whether the player won the point, after this shot or later on (**right**).

Figure 12 shows the result for the test match.

#### 8.3.3. S3: “The Game Speed Is Close to a Second and a Half”

We will analyze S3 through the following query:

**Q3** 
“For each shot, get its duration (the time interval between a shot and the next shot)”.

Using our API, this can be translated as a query that retrieves all shots except the last shot in a point, because the duration is not well defined of these:

def all_non_last_shots(shot):
    return shot.next *# equivalent to: shot.next is not None*
 
attribs = [“hitter.position.x”, “hitter.position.y”, “id”, “duration”]

We can plot the distribution of the duration variable (mean = 1.25 s for the test match), as well as the position of the shots, colored by duration:

q.plot_distribution(“duration”)
q.plot_positions(color=‘duration’)

**Figure 13:**
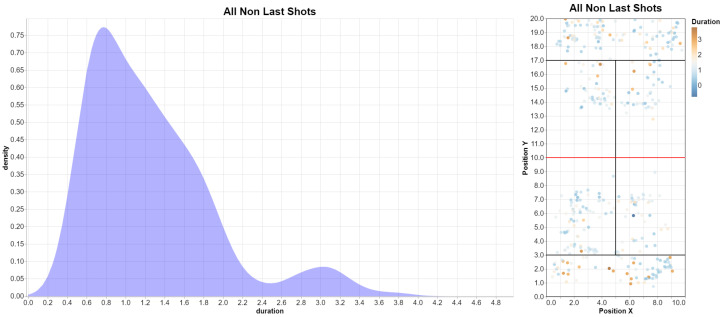
Distribution of the shot duration (s) for a test match (**left**), and position of the players for each shot (**right**), colored by shot duration. One half of the shots had a duration below 1.1 s.

Figure 13 shows the resulting plots. Most of the long shots, as expected, correspond to lobs and passing shots.

#### 8.3.4. S4: “The Serve Should Be a Deep Shot”

Here the query definition is quite simple:

**Q4** 
“Retrieve all serves; for each serve, get the serving player’s position and the serve direction”.

In our API:

@shot_query
def serves(shot):
    return shot.like(“serve”) and shot.next
q = serves(match, attribs)

**Figure 14:**
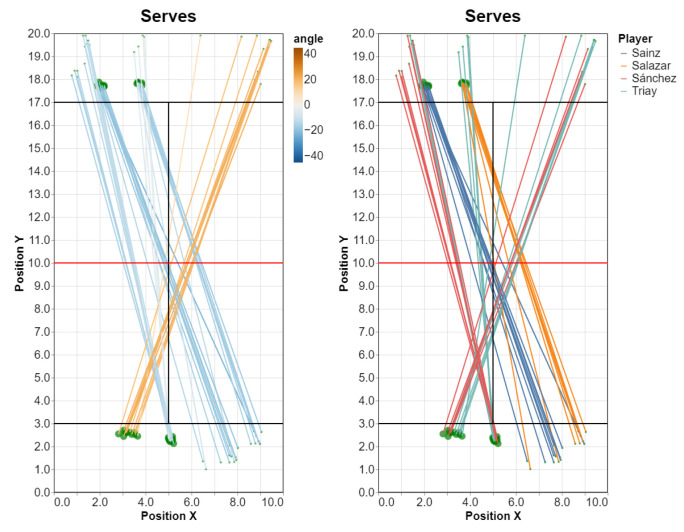
Serve directions, colored by angle (**left**) or by player (**right**). Please recall that, for all plots in this paper, we considered a subset of the points (this is not needed when using the interactive and zoomable plots).

Where attribs list contains the necessary attributes for the analysis. We can plot, for example, serve directions, coloring them either by angle or by player (Figure 14).

q.plot_directions(color=“angle”)
q.plot_directions(color=“player”)

#### 8.3.5. S5: “Serving Player’s Partner Should Be Waiting in the Net Zone”

The query definition is very similar to the previous example, but now we will retrieve (as an attribute) the position of the partner:

**Q5** 
“Retrieve all serves; for each serve, get the position of the partner of the serving player”.

**Figure 15:**
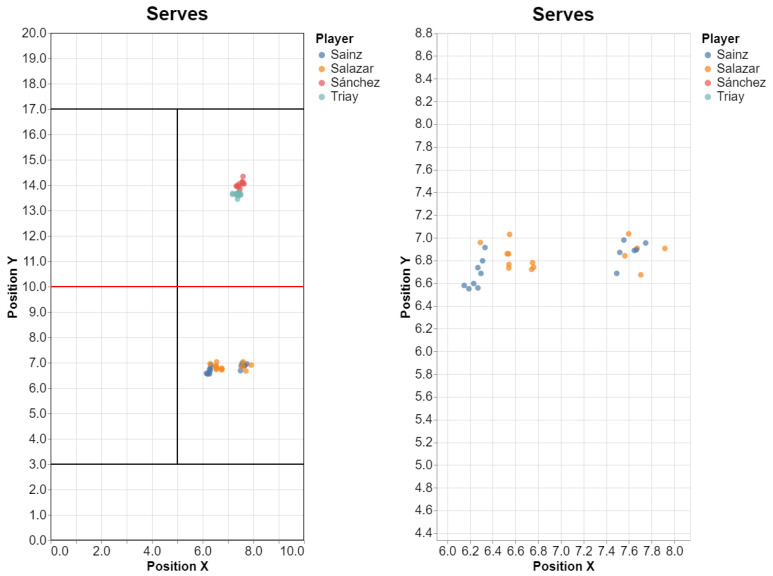
Position of the partner during serves (and a zoom into the clusters on the bottom side).

Using our API, we would use the query definition of the previous example, but we add query attributes to acquire data about the player’s partner (see Figure 15 for the results).

q = serves(match, attribs + [“hitter.partner.last_name”, “hitter.partner.position.x”,
                  “hitter.partner.position.y”])
q.plot_positions(color=‘player’)

#### 8.3.6. S6: “After Serving, the Player Should Move Quickly to the Net”

All the queries so far required data (e.g., the type of shot, the position of the players) at a very specific moment (the time a shot is executed). Now we wish to analyze the movement/paths of the players for some time (e.g., one second immediately after a serve). This means we will have to use a frame_query which can provide data about arbitrary segments of the video.

**Q6** 
“Retrieve all frames immediately after a serve (e.g., for 1 s); for each frame, get the position of the serving player”.

Using our API, we could compare the current frame number with that of the point’s start frame, to filter the frames immediately after a serve (i.e., immediately after the start of the point). See Figure 15 for the results.

@frame_query

def all_frames_after_serve(frame):
    return (frame.time - frame.point.start_time) < 1
 
q=all_frames_after_serve(match, fattribs)
q.plot_player_positions()

**Figure 16:**
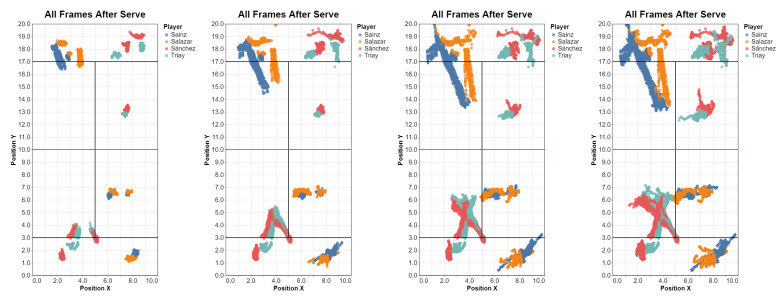
Motion of the four players a after a serve: 0.5, 1.0, 1.5 and 2.0 s. Please note that court-space players’ positions in our test datasets were approximate, with a larger error for players in the top side of the court.

We plot all four players, although of course we could filter only serving players’ paths (Figure 16).

#### 8.3.7. S7: “Players Should Try to Win the Net Zone as Much as They Can”

We will analyze S7 through two queries.

**Q7a** 
“For each point, compute the total time both players were in the net zone, for the team that wins the point, and also for the team that loses the point”.
**Q7b** 
“For each winning shot, retrieve the position (and distance to the net) of the player that hit that ball”.

Using our API, **Q7a** can be translated as

@frame_query

def time_on_net_for_winning_team(frame):
    winner = frame.shot.point.winner
    return winner and frame.distance_to_net(winner.forehand_player) < 4
           and frame.distance_to_net(winner.backhand_player) < 4
 
q1=time_on_net_for_winning_team(match,[“duration”, (“point.id”, “point”)])
q1.sum(“point”) *# Group by point and sum*

where we compute for how long both players of a team are in the net zone (here, 4 m from the net).

Similarly, we can define a query for the losing team, and combine both queries into a single plot:

q1.addColumn(“Team”, “Point Winner”)
q2.addColumn(“Team”, “Point Loser”)
q = concat([q1,q2], “Time on the net”)
q.plot_bar_chart(x=‘point’, y=‘duration’, color=‘Team’)

**Figure 17:**
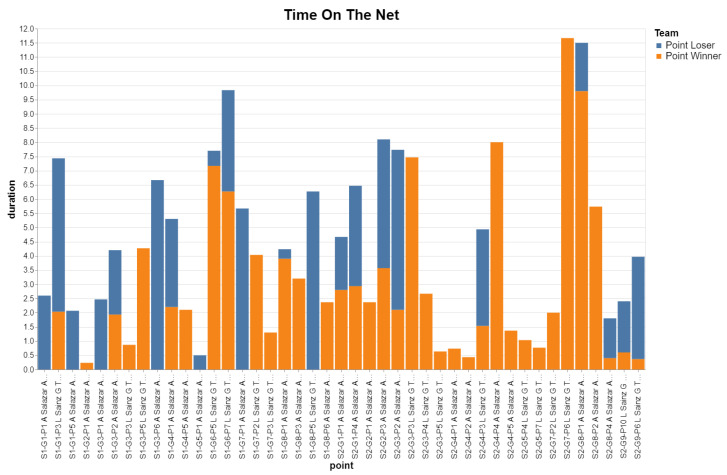
For each point, the bar chart shows the time (s) spent in the net zone (less than 4 m from the net) by the players of the point winning team and the point losing team. Point labels include the game-set-point id of the point, and the team that won that point.

Figure 17 shows the plot we got for our test match. This shows that, for that match, the time on the net for the point winning team was higher (total time: 110.8 s) than for the point losing team (total time: 68.7 s).

Similarly, **Q7b** can be translated as follows:

@shot_query
def winning_shots(shot):
    if not shot.hitter.from_point_winning_team:
      return False  *# not from the winning team*
    return not shot.next or not shot.next.next *# just winning shots*
 
attribs = [“hitter.last_name”, “hitter.position.x”, “hitter.position.y”,
      “frame.frame_number”, (“hitter.distance_to_net”, “distance”)]
 
q = winning_shots(match, attribs)

We can plot the position of the players at the moment they played the winning shot:

q.plot_positions()
q.plot_histogram(“distance”)

**Figure 18:**
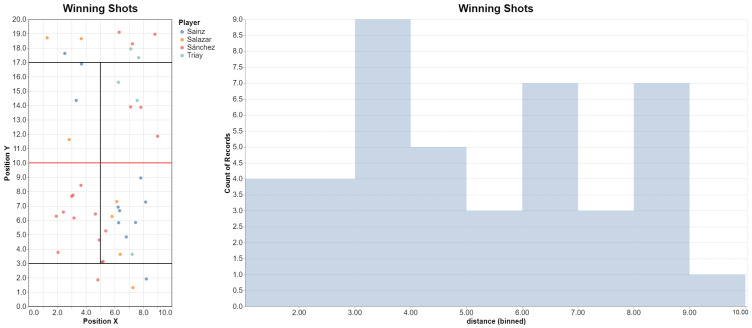
Position of the players (and distance to the net) at the moment they played the winning shot, for our test match.

The resulting plot is shown in Figure 18. Notice that some winning shots were executed from the defense zone; by checking these in the video we observed that in most cases, the opponent made an unforced error when returning these shots (that is, the opponents could hit the ball, but not accurately enough to keep playing the point).

#### 8.3.8. S8: “Distance Covered by the Players”

There are many ways to analyze the distance covered by the players. Here we show just one reasonable option:

**Q8** 
“Retrieve all frames, together with the position of the players and the distance they traversed for each point”.

Using our API, we could just retrieve all frames and plot the players’ positions, colored by name:

@frame_query
def all_frames(frame):
   return True
 
q=all_frames(match, fattribs)
q.plot_player_positions()

**Figure 19:**
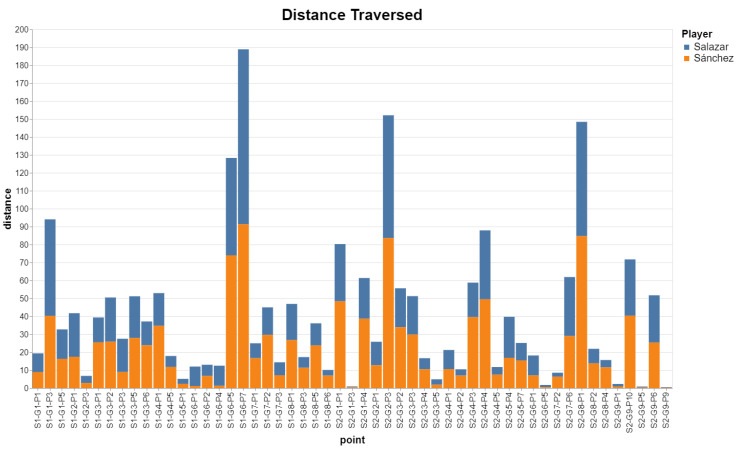
Distance traversed by the two players of a team.

Alternatively, we can compute the traversed distance on a per-frame basis, and then group by point. The example below compares the traversed distance of two players from the same team (Figure 19):

q1=all_frames(match, [(“salazar.distance_from_prev_frame”, “distance”), …])
q1.sum(“point”)
 
q2=all_frames(match, [(“sanchez.distance_from_prev_frame”, “distance”), …])
q2.sum(“point”)
 
q1.addColumn(“Player”, “Salazar”)
q2.addColumn(“Player”, “Sanchez”)
q = concat([q1,q2], “Distance traversed”)
q.plot_bar_chart(x=‘point’, y=‘distance’, color=‘Player’)

#### 8.3.9. S9: “Good Lobs”

The idea here is to quantify the effectiveness of lobs for moving the opponents back to the defensive zone; this can be achieved either by considering the position of the players two shots after the lob, or by checking the type of the opponents’ shot.

**Q9** 
“Retrieve all lobs followed by a defensive shot”.

We can first specify which shots are considered to be *defensive* (we will define a new tag for this, considering the shot types in Table 6):

@shot_tag
def defensive(shot):
  return shot.one_of(“d,r,ad,ar,pld,plr,spd,spr,bpd,bpr,dpa,dpc,dpag,cp,cpld,cplr”)

Now we can retrieve all lobs followed by defensive shots:

@shot_query
def lobs(shot):
 return shot.like(“lob”) and shot.next.like(“defensive”)

We can use query attributes to get additional information about, for example, where the opponents had to return the lob, next.hitter.distance_to_backwall, or the distance to the net of the players two shots after the lob, next.next.hitter.distance_to_net, next.next.hitter.partner.distance_to_net.

#### 8.3.10. S10: “First Volley”

This statement requires identifying the shot sequence *Serve* →*Return*→ *Volley*. We will add additional conditions to check another maxim in padel: if a serve goes to the T, the first volley should direct the ball toward the side wall of the returning player:

**Q10**  “Retrieve all serves directed to the T, followed by any shot, followed by a volley to the side wall of the same player that returned the serve”.

This can be translated as follows, using 2.5 m as the distance threshold:

@shot_query
def  serve_return_volley(shot):
    return shot.like(“serve”) and shot.next.hitter.distance_to_side_wall > 2.5 and
    shot.next.next.like(“volley”) and shot.next.next.next.hitter.distance_to_side_wall < 2.5
    and shot.next.hitter == shot.next.next.next.hitter

where shot is the serve, shot.next is the return, shot.next.next is the volley, and shot.next.next.next is the volley’s return.

### 8.4. Comparison with State-of-the-Art Analysis Tools

We are not aware of any software solution designed specifically for video-based padel analysis. Therefore, we have to consider solutions covering other racket sports. Commercial products typically provide support for the analysis of a fixed, predefined number of variables. It would be unfair to compare our language against these tools, as they simply do not support complex queries (such as Q10).

Fortunately, some video-based, open-source solutions do support arbitrary queries. For example, Lince Plus [38] features the possibility of performing specific analyses with the R language (through RStudio or an integrated console), which in turn is based on DataFrames. Therefore, we believe that the best way to compare our language with the closest state-of-the-art models is to translate some of our test queries to R or Python using raw DataFrame operations.

For the sake of brevity, we will compare the definition of Q10 using Python + Pandas and using our query language.

Using Python + Pandas, in a best-case scenario, Q10 can be defined as follows:

def first_volley(df):
    df = df.reset_index()  *# make sure indexes pair with the number of rows*
    for index, row in df.iterrows():
     if (row[“shot_code”] == “serve” and
        df[“hitter.distance_to_side_wall”][index + 1] > 2.5 and
        df[“shot_code”][index + 2] in [“vd”, “vr”] and
        df[“hitter.distance_to_side_wall”][index + 3] < 2.5 and
        df[“hitter.id”][index + 1] == df[“hitter.id”][index + 3]):
        df.loc[index, “Filter”] = True
 
    return df[ df[“Filter”] == True ]

Notice that we had to loop over the DataFrame (df) rows because the filter involves multiple rows (we are looking for a specific shot sequence). Notice also the use of index offsets, which decreases readability. Finally, we have assumed that the input DataFrame has already been enriched with additional columns (e.g., distance of the player from the net). Otherwise, extra code would be required to compute these columns. This code might not be straightforward if, for example, the filter involves data from other DataFrames (e.g., to relate who played the volley with the team that won the point).

Using our approach, Q10 definition can be written in a more compact and readable way:

@shot_query
def first_volley(shot):
    return shot.like(“serve”) and shot.next.hitter.distance_to_side_wall > 2.5 and
    shot.next.next.like(“volley”) and shot.next.next.next.hitter.distance_to_side_wall < 2.5
    and shot.next.hitter == shot.next.next.next.hitter

## 9. Discussion

Expressiveness was our main priority when designing a domain-specific query language for padel matches. Besides the statements discussed in Section 8, we have been able to write queries to support, refute or analyze all statements about padel strategies under the only assumption that the queries involve concepts captured by the tabular data. This is not a limitation of the language, but of the sensing technology to generate the input datasets. For example, our dataset has no information about shot effects (backspin, topspin, slice).

We have observed that being able to *navigate* across temporal concepts (from shots to frames, frames to shot, from a shot to the next…) largely simplifies the readability and compactness of the queries. As shown in many examples, the separation between the query definitions (“retrieve all volleys”) and query attributes (“get the player’s position”) facilitates code reusability. User-defined tags and properties also provide a simple mechanism to include new concepts (“deep lob”) that further simplify the task of writing queries in a language close to that of coaches.

The output of a query is a QueryResult object that keeps internally a Panda’s DataFrame object. As shown in many examples, this class provides methods for essential plots. Supported plots include scatter plots for players’ positions on an overhead view of the court, as well as bar charts and histograms. More complex plots can be obtained by accessing directly the query output and using any visualization tool (we used Vega-Altair as well as HoloViz’s hvPlot).

Our approach though has some limitations. The flexibility of using a programming language, with arbitrary predicates on the query definitions, and arbitrary expressions on query attributes, comes at the price of raising the entry barrier for sports analysts to use the tool, since some Python skills are needed to write new queries. Although we believe that minor edits to the query definitions are doable with little Python knowledge, the main problem is the interpretation of potential syntax errors. Despite this, we believe that the queries using the proposed API are more readable than alternative methods.

Although the focus of this paper is the query language and not how the input data have been obtained, the availability of large datasets including accurate data about many matches would certainly influence the impact of this work. On our test dataset, the most relevant issue was the accuracy of the positional data, which was questionable for players on top of the video, and also when jumping, since the perspective correction we apply to move from image space to court space coordinates assumes that the feet are on the floor. Advances in video tracking and pose estimation techniques, or the use of multi-camera approaches, would improve the quality of the data and thus the reliability of analysis tools.

## 10. Applications

The proposed API has multiple practical applications, as it simplifies writing queries and facilitates the exploratory analysis of padel matches.

At a professional level, the tool speeds up the analysis of many variables and their relationship. For example, the tool can be useful in the following tasks:

- Determine the game profile in professional padel, considering variables such as the number of games, number of points, average duration of games and points, time interval between shots, number of winning points, and number of unforced errors [5,44].
- Analyze the frequency and success rate of the different technical actions (types of shots, their direction and speed) according to the in-game situation (preceding technical actions, position, and speed of the partner and the opponents).
- Analyze the distance covered by the players, their positions, displacements, and coordinated movements, and relate them with the other variables [45].
- Analyze how all the variables above vary between women’s matches and men’s matches.
- Analyze how other external factors (e.g., outdoor match) might affect the variables above.
- Retrieve specific parts of the video (e.g., certain shot sequences) to quickly analyze visually other aspects not captured by the input tabular data.

At an amateur level, coaches and padel clubs might offer the opportunity to record the videos of the training sessions to further analyze them. For example,

- Compare the technical actions adopted by the trainee in particular scenarios against those adopted by professional players.
- Show trainees specific segments of professional padel videos to provide visual evidence and representative examples of strategic recommendations.
- If multiple videos are available, compare the different variables defining the game profile of a trainee with those of other amateur or professional players.
- Help to monitor the progress and performance improvement of the trainees.

## 11. Conclusions and Future Work

In this paper we have presented a domain-specific query language, in particular a Python API, to analyze tabular tracking data on padel matches. We foresee that, as tracking software becomes more robust and accurate, more specialized tools will be required to fully benefit from these data.

We have focused on padel because it is arguably the racket sport where tactics are more complex (due to the enclosing balls and ball bounces) and have a rich variety of technical actions. The fact that there is a large body of publicly available videos of padel matches, most of them from the de facto camera standard [21], also facilitates the digitization of matches. Despite this, we believe that most ideas of the query language can be applied to other racket sports such as tennis. As future work, we plan to evaluate the readability of the queries (and the ease of editing them) for different user profiles. We also plan to build a visual front-end to allow coaches with absolutely no programming experience to create their own queries. Similarly, an interesting avenue is to explore the use of GUI-based software wizards to guide users through a sequence of steps to create complex queries.

## Figures and Tables

**Table 1:** Example of raw tabular data for padel *points*. The time units for the first three columns are frames. The Winner team is identified by the top/bottom position on the video.

Start (f)	End (f)	Duration (f)	Duration (s)	Winner	Points A	Points B	Top Left	Top Right	Bottom Left	Bottom Right
13,606	13,820	214	7.1	B	0	15	L Sainz	G Triay	A Sánchez	A Salazar
14,093	14,785	692	23.1	T	15	15	L Sainz	G Triay	A Sánchez	A Salazar
15,332	16,204	872	29.1	T	30	15	L Sainz	G Triay	A Sánchez	A Salazar
16,932	17,004	72	2.4	B	30	30	L Sainz	G Triay	A Sánchez	A Salazar
17,378	17,661	283	9.4	B	30	40	L Sainz	G Triay	A Sánchez	A Salazar

**Table 2:** Example of raw tabular data for padel shots. The shot type uses the classification proposed in [5]. The Lob column contains a Boolean that indicates whether the shot is a lob.

Frame	Player	Shot Type	Lob
13,604	TL	Serve	F
13,633	BR	D	T
13,656	TL	B	F
13,676	BR	D	F
13,696	TL	VD	F

**Table 3:** Example of raw tabular data for the frames of a video. Players are referred to with their location in the video at serve time (e.g. TL means top-left player). Positions are given in image space (*i*, *j* are in pixels) and court-space (*x*, *y* are in meters).

Frame	TL i	TL j	TR i	TR j	BL i	BL j	BR i	BR j	TL x	TL y	TR x	TR y	BL x	BL y	BR x	BR y
13,614	478	202	785	266	401	554	911	553	1.89	18.53	7.49	13.60	2.50	1.60	7.87	1.66
13,615	477	202	785	266	401	555	912	554	1.87	18.52	7.49	13.60	2.50	1.59	7.88	1.62
13,616	477	203	785	266	400	553	914	555	1.89	18.43	7.49	13.60	2.49	1.63	7.89	1.60
13,617	479	204	785	266	399	550	915	556	1.94	18.35	7.49	13.59	2.47	1.70	7.90	1.57
13,618	480	206	785	266	398	549	918	556	1.96	18.21	7.49	13.59	2.46	1.74	7.93	1.58

**Table 4:** Translation of the application domain concepts to Python language.

Application Domain Concepts	Python Language Translation
Match, Set, Game, Point, Shot, Frame	Python classes corresponding to temporal play units.
First name, Last name, Gender	Python class properties referring to a player.
Start, End, Duration	Python class properties of a play unit.
Winner, Player	Python classes (Team, Player).
Score	Python class property.
Query definition	Python function (decorated).
Query type	Python function decorator.
Query filter	Python function definition.
Query name	Python identifier.
Query predicate	Body of a Python function that returns a Boolean value.
Query execution	Invocation of the function identified by the Query name.
	The function takes two parameters: the scope and the attributes.
Query scope	Python expression that evaluates to a match or a collection of matches.
Query attributes	Python list containing strings representing Python expressions.
Query result	Python class that holds the output table.

**Table 5:** Query types supported by our query language.

Query Type	Python Decorator	Output DataFrame
Match query	@match_query	One row for every match that meets the query definition.
Set query	@set_query	One row for every set that meets the query definition.
Game query	@game_query	One row for every game that meets the query definition.
Point query	@point_query	One row for every point that meets the query definition.
Shot query	@shot_query	One row for every shot that meets the query definition.
Frame query	@frame_query	One row for every frame that meets the query definition.

**Table 6:** Shot types with native support in our API. These shot types are based on the classification proposed in [5] (except the serve). Each row corresponds to a shot type. We provide the shot code, its Spanish expansion [5], and some equivalent strings to represent them in queries.

Shot Code	Spanish Name	English Translations
“s”	“servicio”	“serve”
“d”	“derecha”	“drive“, “forehand”
“r”	“revés”	“backhand”
“ad”	“alambrada derecha”	“right mesh“, “right fence”
“ar”	“alambrada revés”	“left mesh“, “left fence”
“pld”	“pared lateral de derecha”	“side wall drive”
“plr”	“pared lateral de revés”	“side wall backhand”
“spd”	“salida de pared de derecha”	“off the wall forehand”
“spr”	“salida de pared de revés”	“off the wall backhand”
“bpd”	“bajada de pared de derecha”	“off the wall forehand smash”
“bpr”	“bajada de pared de revés”	“off the wall backhand smash”
“dpa”	“doble pared que abre”	“double wall opening”
“dpag”	“doble pared que abre con giro”	“double wall opening with rotation”
“dpc”	“doble pared que cierra”	“double wall closing”
“cp”	“contrapared”	“back wall boast”
“vd”	“volea de derecha”	“drive volley“, “forehand volley”
“vr”	“volea de revés”	“backhand volley”
“b”	“bandeja”	“defensive smash”
“djd”	“dejada”	“stop volley“, “drop shot”
“r1”	“remate”	“smash”
“r2”	“finta de remate”	“fake smash”
“r3”	“remate por 3”	“smash out by 3”
“r4”	“remate por 4”	“smash out by 4”
“cd”	“contra-ataque de derecha”	“forehand counter-attack”
“cr”	“contra-ataque de revés”	“backhand counter-attack”
“cpld”	“contrapared lateral derecha”	“right wall boast”
“cplr”	“contrapared lateral izquierda”	“left wall boast”

## Data Availability

The source code of a free implementation of the API will be released upon acceptance. The test video is publicly available at https://youtu.be/7s55wB9dR78 (accessed on 1 December 2022).

## Supplementary Materials

The following supporting information can be downloaded at: https://www.mdpi.com/article/10.3390/s23010441/s1, Video S1: Interactive Demo.

## Appendix A. Implementation Details

Our API extensively uses Python’s function decorators, such as @shot_tag and @shot_query. A decorator is a mechanism of the Python language for defining higher-order functions, that is, functions that take another function and extend its behavior. For example, when we add the @shot_tag decorator to a Boolean function deep_lob, in fact, we are creating a higher-order function that, when invoked on a match, traverses all shots, evaluates the Boolean function deep_lob on each shot, and if the function evaluates to true, adds a new tag to the shot called “deep_lob”. This mechanism is convenient because it moves a large part of the boilerplate code from the tag/property definition to the internal implementation of the API.

The same applies to query decorators such as @frame_query. Adding this decorator to a Boolean function, creates a higher-order function that, when invoked on a match, traverses all frames, evaluates the Boolean function on each of frame, and adds the user-defined attributes to the output if the function evaluates to true.

The evaluation of query attributes (which can be arbitrary Python expressions) is also handled by the higher-order function created by the query decorators. The internal code takes care of evaluating the attributes (using Python’s eval function), adding new columns to the output DataFrame, and filling them with the data.
